# Resilience to Climate-Induced Disasters and Its Overall Relationship to Well-Being in Southern Africa: A Mixed-Methods Systematic Review

**DOI:** 10.3390/ijerph15112375

**Published:** 2018-10-26

**Authors:** Joseph K. Kamara, Blessing J. Akombi, Kingsley Agho, Andre M. N. Renzaho

**Affiliations:** 1 School of Social Sciences and Psychology, Western Sydney University, Sydney, Locked Bag 1797, Penrith, NSW 2751, Australia; B.Akombi@westernsydney.edu.au (B.J.A.); Andre.Renzaho@westernsydney.edu.au (A.M.N.R.); 2 School of Sciences and Health, Western Sydney University, Locked Bag 1797, Penrith, NSW 2751, Australia; k.agho@westernsydney.edu.au

**Keywords:** resilience, recurrent disaster, drought, rural, subsistence farming, southern Africa

## Abstract

The available literature suggests that natural disasters, especially droughts and floods, were occurring in southern Africa in the early 1900s. However, their frequency and intensity increased during the 1980s. The aim of this systematic review was to assess the relationship between resilience to droughts and people’s well-being in southern Africa. A combination of keywords was used to search the following 13 electronic bibliographic databases: Africa Journal Online (AJOL), MEDLINE, Academic Search Complete, Environment Complete, Humanities International Complete, Psychology and Behavioral Sciences Collection, PsycINFO, Embase, Scopus, Web of Science, Applied Social Science Index and Abstracts, ProQuest Central, and CINAHL. Relevant websites were also searched and potential studies for inclusion were downloaded in an EndNote database and screened for eligibility using pre-determined criteria. Quality assessment of the studies was undertaken using the Joana Briggs Qualitative Assessment and Review Instrument, the National Institutes of Health (NIH) checklist, and the Authority, Accuracy, Coverage, Objectivity, Date, Significance (AACODS) checklist. Resilience and well-being scales used in the studies for inclusion were also assessed using pre-defined criteria. Nineteen studies met the inclusion criteria. Poverty alleviation policies were important in strengthening resilience and well-being outcomes. Resilience and well-being were connected by old age, gender, race, adaptive farming and livelihoods diversification, security, and knowledgeability. Resilience and well-being outcomes were advanced by the synergistic effect of household, community and governance level capacities encapsulated in knowledgeability. This systematic review is critical to improving southern Africa context-specific resilience, and well-being policies and interventions.

## 1. Introduction

Disasters have increasingly become regular and omnipresent, with devastating effects on humans and ecosystems. In this study, we define a disaster as a catastrophe resulting in injury, loss of life, and social and economic disruption that exceeds the coping capacity of the affected people and the ecology [1,2]. There are two forms of disasters: natural and human-induced. Naturally occurring disasters can be classified into five groups, namely, geophysical: earthquakes, landslides, tsunamis, and volcanic activity; hydrological: avalanches and floods; climatological: extreme temperatures, droughts, and wildfires; meteorological: cyclones and storms/wave surges; and biological: disease epidemics such as Ebola and insect infestation such as locust invasions [3]. In contrast, human-induced disasters include armed conflicts and wars, famine, ethnic conflicts and displaced populations, industrial and transport accidents, environmental degradation such as pollution, as well as anthropogenic intentional hazards such as terrorism or weapons of mass destruction [3,4].

People cannot effectively stop natural disasters such as droughts but they can mitigate disaster risks and reduce their negative impacts through information sharing, behaviour change, effective governance and technological innovations [1]. While humans cannot stop natural disasters, frameworks such as that proposed by Birnbaum and colleagues [5], suggest that we can create a resilience to disasters by identifying and experiencing disaster risks, and we can learn from them to predict the future and selectively choose the most likely risks to address. The framework further posits that choosing the risks to address accompanied by the identification of existing resilience standards and their evaluation; identification of gaps and commensurate interventions; preparing work plans, execution and appraisal collectively strengthen disaster resilience capabilities [5]. Similarly, the Sendai framework for disaster risk reduction 2015–2030 proposed that nations can create and strengthen disaster resilience capabilities by gaining an understanding of disaster risk; enhancing disaster risk governance; investing in disaster risk reduction and; building back better after a disaster event [1]. The Sendai framework for disaster risk reduction succeeded in the Hyogo Framework for Action (HFA) 2005–2015 which sought to build the resilience of nations and communities to disasters. The HFA came after the Yokohama Strategy for a Safer World which provided the basis for the 1999 international strategy for disaster reduction [6].

There is no universal definition of resilience. Attempts to define resilience have confined it to the parameters for specific study areas that seek to investigate resilience to what, how, and by whom [7]. For this systematic review, resilience is defined as the capability to leverage the environment to expect, withstand, recover, and adjust to disaster-induced distress [8]. The capabilities exist in isolation or in tandem at the individual, household, and collective level. Therefore, we examine resilience at all levels so as to frame it within the collective nature of the study area’s way of life. This approach enables relevant contextually and culturally appropriate study outcomes. It is anticipated that recurrent drought leaves some form of adaptive capabilities at the various levels which makes resilience to drought an important subject of interest. We define well-being as an agreeable state characterised by the absence of ill health.

This systematic review focuses on resilience to climate-induced disasters, especially drought in southern Africa for various reasons. Foremost, southern Africa experiences recurring droughts. Evidence suggests that southern Africa experienced droughts in the early 1900s [9,10,11]. However, in the 1980s, there was a remarkable spike in the frequency, intensity, and impact of droughts across the region [12,13]. Since then, naturally occurring disasters, especially droughts that are induced by the El Nino southern oscillation, have occurred almost every two years [10,14,15]. Recurrent droughts devastate livelihoods and induce humanitarian interventions that help communities cope, thus, promoting a false and unsustainable sense of defiance against droughts [16]. Humanitarian interventions are critical enablers of recovery but their prescriptive nature and externality often ignore inherent capacities such as the traditional knowledge of the communities they seek to help [17,18]. Evidence suggests that traditional knowledge and culture are vital and enabling elements of disaster resilience [19]. Governments and their partners across the region have a responsibility to leverage inherent capacities to build and strengthen resilience, especially in response to recurrent threats such as droughts which have become a ‘new normal.’ However, the stakeholders are yet to work out a comprehensive approach to building drought resilience. The synthesised information from this systematic review will provide the foundation that informs the approach.

Secondly, unlike the rest of sub-Saharan Africa, which also experiences droughts, southern Africa countries are classified as middle-income development status. This development status is expected to be leveraged in building and demonstrating resilience to disasters. Moreover, recurring droughts in the region are no longer unexpected but a reality. Countries in other sub-Saharan Africa regions are of a low-income development status and are expected to have less disaster coping capacities compared to southern Africa. Lastly, natural disasters in other sub-Saharan Africa regions attract quick donor attention and large funding compared to southern Africa [20,21,22]. The differences enumerated above are contextual to the region and affect the timely and effective disaster response. This uniqueness has compelled us to confine our systematic review to the southern Africa region, anticipating context-specific findings that will inform the region’s disaster management strategies and policies.

Evidence suggests that southern African governments have not sufficiently prioritised drought resilience building; instead, they are caught in cyclical responses to recurring droughts [23,24]. This could be attributed to underlying vulnerabilities such as a weak governance and institutional capacity, accompanied by a high inequality, poverty, and environmental degradation [24,25,26]. Drought response strategies usually tend to be humanitarian in nature, which is critical in disaster recovery of the predominantly rural and subsisting communities. Recovery deals with the planning and allocation of resources to restore capabilities lost or degraded by a disaster event. During emergency operations, humanitarian assistance focuses on emergency needs and often neglect building and strengthening resilience capabilities. More so, because political interference with local processes and the long time required implementing resilience interventions make it difficult for humanitarian responses to focus on resilience. The underlying vulnerabilities such as weak governance, land degradation, and poverty remain unaddressed, yet they are critical to resilience building. For example, over 70% of the population in the region is subsisting and rural based, and almost half of these live on less than one US dollar a day [27]. Drought-combating strategies that do not address rural poverty and inequality affecting such a large proportion of the population would be ineffective. Such interventions create a trajectory of communities bouncing back to their pre-disaster state of poverty and vulnerability. Nonetheless, southern Africa has exhibited some form of drought resilience activities such as land reclamation and rangeland management in Lesotho, crop and livestock diversification in Namibia and South Africa. Another example is the regional vulnerability assessment and analysis program (RAAV) which monitors ongoing integrated food security classification and vulnerability as well as member states’ disaster management capabilities [28,29,30,31].

There are gaps in the research on how resilience to droughts relates to the well-being of rural subsistence communities. The gaps are perpetuated by the lack of adequate contextual resilience measures to inform resilience policies and interventions. Generating evidence to bridge this gap has the potential to mitigate the negative impact of droughts on rural communities and help them recover in the most rapid and sustainable way. In addition, it will enable governments and humanitarian and development agencies to reduce expenditure on repetitive and costly drought interventions. The evidence will also inform the implementation of the United Nation’s Sustainable Development Goals (SDGs) in the region, specifically those that address poverty (SDG1), hunger (SDG2), and health and well-being (SDG3) [32]. Therefore the purpose of this study is to assess the relationship between resilience to drought and well-being; and to identify gaps where further research is needed to inform a standard and contextual measure of resilience. However, resilience has been conceptualised differently and there is no single definition, hence, it was important to assess the suitability of the instruments used to measure resilience.

## 2. Methods

This systematic review was planned, implemented, and reported in accordance with the Preferred Reporting Items for Systematic Reviews and Meta-Analyses (PRISMA) guidelines (S 1). The PRISMA statement is a checklist with 27 essential items for ensuring reporting transparency [33]. Details of this systematic review’s methods were registered by the PROSPERO international prospective registry for systematic reviews, reference CRD42017064396 [34]. Additionally, a protocol has been published in BMC Systematic Reviews [16].

### 2.1. Search Strategy

A librarian experienced in systematic reviews assisted in the development of the search strategy (see Acknowledgements). Thereafter, a comprehensive search was conducted from 1 January 1980 through to 10 August 2018. The following 13 electronic bibliographic databases were searched: Africa Journal Online (AJOL), CINAHL, MEDLINE, Academic Search Complete, Environment Complete, Humanities International Complete, Psychology and Behavioral Sciences Collection, PsycINFO, Embase, Web of Science, Applied Social Science Index and Abstracts, ProQuest Central, and Scopus. Additionally, multi-disciplinary databases and key organisational websites such as FAO, Google Scholar, Global Health Library, OECD, the World Bank, IFRC, USAID, and World Vision were searched. The following combination of search terms and keywords was used in the search:

Resilien* OR adapt* OR coping OR adjustmen* OR coheren*)

AND (drought* OR disaster*) AND (Well-being* OR wellbeing* OR disparit* OR status)

AND (Southern Africa* OR Botswana* OR Lesotho* OR Namibia* OR Swaziland* OR South Africa*)

Search terms varied slightly for each database. Studies were extracted into an EndNote library where they were subjected to further screening, as explained under ‘Data extraction and quality assessment’.

### 2.2. Types of Participants

This systematic review is not limited by the participants’ age, gender, social status, or ethnicity. Participants are drawn from the Southern Africa region where a large proportion of the region’s population is rural, subsists on agriculture, and is faced with recurrent food shortages due to droughts [16].

### 2.3. Inclusion and Exclusion

Due to the nature and purpose of the study, we established wide inclusion criteria to broaden the literature coverage. This was mainly because disaster resilience is increasingly becoming a policy issue for governments across southern Africa. Therefore, studies were included if they (1) were published between January 1980 and 10 August 2018; (2) were peer-reviewed articles, dissertations, books and book chapters, working papers, technical reports, discussion papers, and conference papers; (3) were written in English and their full texts were available and accessible as we did not have the logistical and financial capacity to search for, retrieve and translate literature published in languages other than English; (4) measured resilience and its relationship to well-being; (5) were conducted in the southern Africa region. Reference lists of studies included were perused to identify relevant studies; those that met the criteria were included in the review. We define southern Africa as a geographical region comprised of Botswana, Lesotho, Namibia, South Africa, and Swaziland, as delineated by the United Nations geographical regions [35]. A search log was developed and used for accountability and transparency.

Studies were excluded if (1) they were carried outside the stated time frame; (2) conducted in countries other than the southern Africa region; (3) published in languages other than English; (4) were reviews, editorials, letters to editors, and opinion pieces; (5) and/or did not assess the relationship between drought resilience and well-being.

#### 2.3.1. Data Extraction and Quality Assessment

Studies retrieved from databases were imported into an EndNote library and screened by title to eliminate duplicates. This was followed by a screening of the study abstracts to determine relevance. Thereafter, full texts of the remaining studies were read for eligibility and studies that met the inclusion criteria were retained. The extraction and appraisal of retrieved studies was conducted by one author (JKK) and independently reviewed by a second author (BJA). The two authors perused the reference lists of the retained studies to identify additional relevant studies. A third reviewer (AR) adjudicated the differences that emerged in the selection of the final studies for inclusion.

Piloted forms informed by the Cochrane Handbook for Systematic Reviews and the Joanna Briggs Institute (JBI) reviewers manual, 2014 edition, were used in the extraction of quantitative and qualitative data [36,37]. Extracted studies were identified by their author, year of publication, country, characteristics, design and data collection methods, setting, objectives, resilience outcome measures, instrument strength, and well-being (Table 1).

The quality assessment of the studies included involved two parts. Foremost was the analysis of the methodological quality of the studies. This enabled the evaluation of limitations and appropriateness of the studies’ methods in addressing their research questions and objectives, and outcomes. Thereafter, we assessed the psychometric properties of the tools used in the studies.

#### 2.3.2. Methodological Quality Assessment

We assessed the studies’ designs, methods of data collection and analysis, selection bias, integrity, confounders, and reporting and summarised the findings as high medium or low using the appropriate tools. We appraised the dependability of the qualitative studies using the Joanna Briggs Institute Qualitative Assessment and Review Instrument (JBIQARI) [37]. The JBIQARI is an effective tool for assessing the risk of bias and a study’s methodological quality. The results were assessed and scored as <4 points (low), 5–7 points (medium), or 8–10 points (high) (Table S2). Thereafter, we appraised the quality of grey literature using the Authority, Accuracy, Coverage, Objectivity, Date, Significance (AACODS) checklist [38]. The AACODS tool has been widely used in the quality assessment of grey literature by various studies and was found to produce rigorous results [39,40]. The use of AACODS enabled us to grade the studies as high (24–32 points), medium (15–23 points) or low (≤14 points) (Table S3).

We did not encounter peer-reviewed studies that were experiments, quasi-experiments, or evaluations of interventions as anticipated in the protocol. Therefore, we used the National Institutes of Health (NIH) checklist to assess peer-reviewed observational studies that were quantitative. The NIH checklist measures 14 unique criteria to assess the internal validity of studies [41]. Studies were considered as good if they met the 10–14 criteria, fair if they met the 5–9 criteria and poor if they met the <4 criteria (Table S4). A high-quality rating implies a low risk of bias and vice versa [41]. Emerging evidence suggests that the NIH checklist is a robust tool for assessing risk bias in observational and cross-sectional studies [42,43,44].

We used a combined framework informed by Nukunu, Tolley, and colleagues for the thematic analysis of qualitative studies [45,46]. This enabled the identification of common threads of how resilience and well-being were conceptualised in the studies. The process involved first identifying patterns in the data. The second step was to search for relationships, interactions, and connections between patterns and themes. The third step was to ponder on the several meanings given to the same terms, and the fourth step involved giving meaning and significance to patterns observed in the data. The fifth step was to explore reasons to support the patterns and, lastly, the sixth step involved examining the relevance of emerging patterns to our research question.

#### 2.3.3. Psychometric Properties Quality

With the exception of qualitative studies, the psychometric properties of the rest of the study scales were assessed for content validity, reliability, criterion validity, and construct validity using the Cyril and colleagues framework [47]. The components were chosen because of the breadth of reach in determining the strength of psychometric properties. For example, content validity analyses the extent to which a specific measure addresses all aspects of a given construct. We undertook the content validity to examine the extent to which the different facets of the scales used in the studies measured resilience. This involved assessing whether the study instruments were informed by a literature review, a panel of experts and empirical studies, and whether the target study audience had the opportunity to review the instruments. For each facet addressed, a point was awarded. We assessed the instrument reliability to establish if the scales used in studies consistently measured resilience. This was achieved by assessing whether the studies reported on the use of Cronbach Alpha and the test-retest measures. The Cronbach Alpha were scored as unacceptable <0.50 (0 points); poor ≥0.50 and <0.70 (1 point); acceptable ≥70 and <0.80 (2 points); and good ≥0.80 (2 points). The results of the test-retest were assessed as poor <0.40 (0 points); fair ≥0.40 and <0.60 (1 point); good ≥0.60 and <0.75 (2 points); and very good ≥75 (3 points).

We assessed the criterion validity of the scales to test the extent to which the latter related to well-being outcomes. This was done by establishing the correlation indices between resilience scores with well-being outcomes determined by grading the indices, whereby ≥0.70 meant a strong linear relationship (3 points), 0.50 meant moderate linear relationship (2 points), 0.30 meant a weak linear relationship (1 point), and 0 meant no linear relationship (0 points). We used the construct validity to assess if the instruments actually measured resilience, to what extent they did, and to identify the structure in the association between resilience subscales [16]. We examined whether the study instruments were subjected to exploratory and/or confirmatory factor analyses and whether the least factor analysis threshold was met in the subscales. This was done by determining if the extracted factors explained ≥50% of the variance; each extracted factor had at least three items; each variable loaded strongly on only one factor (≥0.35) and had two or more strong loadings (≥0.70); and was based on at least 10 cases per variable. For each criterion met, a point was awarded. The total psychometric properties scale oscillated from 0 to 17 (Table 2). Study instruments were graded as poor (<4 points), acceptable (5–9 points), good (10–13 points), or very good (>13 points) (Table S5).

### 2.4. Data Analysis

We did not carry out a meta-analysis because the retained studies were mainly observational by design and heterogeneous. Instead, we provide a narrative summary of the findings based on tables of ratings and frequencies. Several studies including systematic reviews have successfully used this approach to present their findings [47,48,49]. Nonetheless, an inductive thematic analysis of the qualitative studies’ findings was carried out to identify resilience and well-being themes. The analysis was iterative and involved reading the retained studies to become familiar with their content, the coding of the retained studies’ findings, grouping the codes into common areas to create potential themes, reviewing the themes to generate a thematic map, defining and naming the themes, and linking themes back to the review purpose to generate insight on resilience and well-being. A similar approach to qualitative data analysis in systematic reviews was proposed by Thomas and Harden [50].

## 3. Results

**Figure 1:**
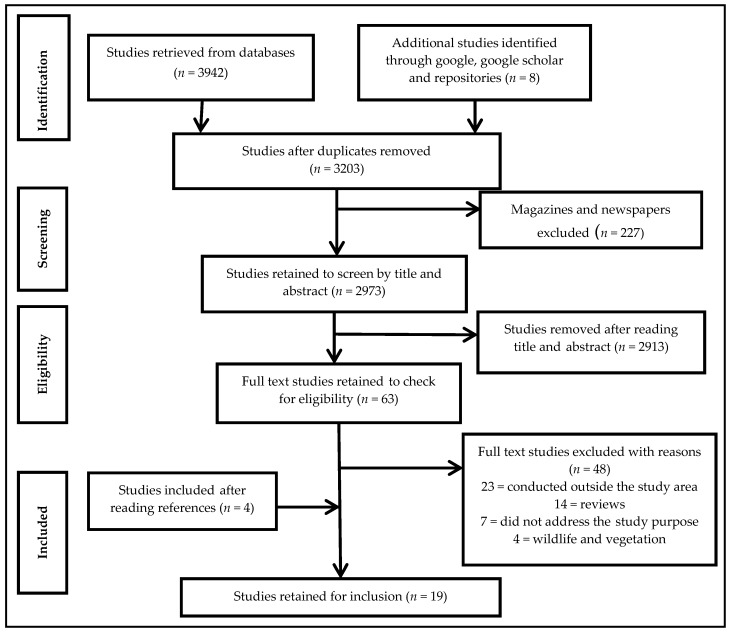
The Preferred Reporting Items for Systematic Reviews and Meta-Analyses (PRISMA) flow diagram.

We report our findings within the confines of the Preferred Reporting Items for Systematic Reviews and Meta-Analyses (PRISMA) guidelines [30]. Our search yielded 3950 studies. Our criteria excluded duplicates (*n* = 747) and magazines, and newspapers (*n* = 227). Screening of titles and abstracts excluded 2913 studies. The full texts of the remaining 63 studies were retrieved and read for eligibility and relevance, which led to a further exclusion of 48 studies. Fifteen remaining studies (*n* = 15) met our inclusion criteria. Reference lists of studies retained were screened and yielded four (*n* = 4) more studies, resulting in a total of 19 studies included in this systematic review (Figure 1).

### 3.1. Study Characteristics

The sample sizes of the retained studies varied from 10 to 3324. Six of the studies used qualitative methods, two applied quantitative methods and the remaining studies used mixed methods. The rural farming communities were the predominant study population except for two studies, one of which focused on drought subject matter experts and the other on anthropometry in children <5 years [17,64].

**Figure 2:**
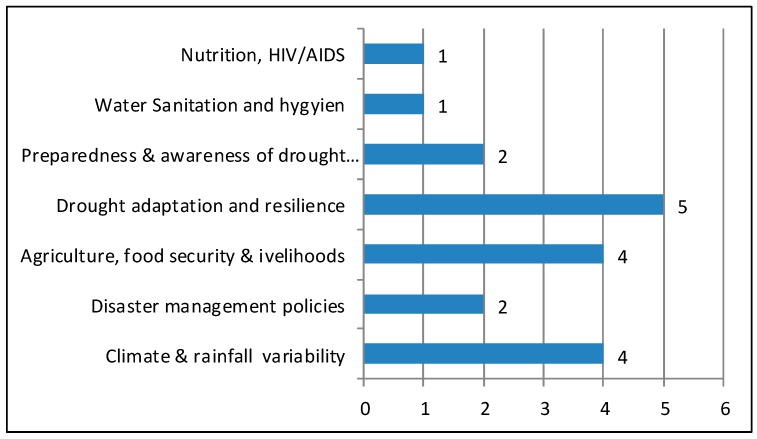
The variability of the studies’ objectives.

Included studies had a range of objectives, from rainfall and climate variability to food security, coping, and adaptation (Figure 2). Nonetheless, all studies aimed to demonstrate the relationship between drought resilience and well-being across southern Africa.

### 3.2. Summary of Findings

Our findings suggest that the most prevalent determinants of drought resilience were political and governance, indigenous and local knowledge, community and household capacities.

#### 3.2.1. Political and Governance Capacities

Eight of the nineteen studies [17,28,30,54,55,56,58,62] examined the political and governance determinants of drought management and response. These studies observed that local politics and governance determined the nature of drought response and resilience outcomes. For example, Van Riet found political and historical legacies such as the 1913 Land Act and tenure racialised farming into predominantly well-resourced white commercial farming and poorly resourced black subsistence farming [30]. The study observed that drought compounded environmental degradation, job and crop losses, as well as low-income levels. However, black subsistence farmers were negatively affected more than their commercial counterparts. Subsistence farmers relied on rudimentary coping mechanisms such as old age pensions, community-saving schemes and burial societies, and sought temporary grazing rights from neighbouring traditional leaders. Burial societies are informal community-saving schemes where members make periodical financial contributions that are drawn from to facilitate funerals. Subsistence farmers experienced deprivation, hunger, helplessness, and structural barriers such as inaccessibility to resources and agricultural land. Commercial farmers owned the best agricultural land and in large quantities, with multiple private water sources such as boreholes. They also enjoyed government subsidies prior to the 1990s in order to support their farming activities. Farmers unable to sustain farming without subsidies left farming for non-farming occupations.

A similar politicisation of farming along the racial divide was observed in a study of commercial and communal farmers in North-West province, South Africa [62]. It was observed that commercial farming was dominated by white farmers to whom farming was a profitable business. They proactively understocked and destocked early enough before the drought conditions worsened to optimise their returns. They also had access to vast pasture land that enabled rotational grazing and reared sheep to supplement income from cattle sales during droughts. During the drought, commercial farmers earned more livestock from their communal counterparts, who rented the former’s pasture land and paid with livestock. The commercially viable farming business enabled the diversification of livelihoods into off-farm business ventures to reduce their drought exposure risk. Black communal farmers relied on unconventional approaches. For example, they shared limited food crops with livestock, allowed livestock to be undernourished in anticipation for recovery after the drought, and combined cattle rearing with goats, which required less water and pasture but was of less economic value. Communal farmers sought wage-based incomes on commercial farms and other businesses but encountered fewer employment opportunities, which affected their purchasing power as well as their food and nutrition needs. Interestingly, commercial farmers were motivated by profit while their communal counterparts practised livestock farming as a cultural value. Both farmer groups highlighted the absence of social support to cope with the negative effects of droughts [62].

However, this claim of lack of social support was contradicted by the rainfall variability study. This study cited social cohesion and support as tenets of the farmers’ resilience to frequent droughts that had devastated farming livelihoods [58]. The study participants claimed that persistent drought led to crop and livestock losses, hunger, tiredness, sickness, indebtedness, and reliance on welfare. However, out of their misery emerged intra-community collective action that enabled government intervention with extension services. The services included the fusion of drought-tolerant indigenous livestock and crop breeds with new varieties for increased productivity. Other government schemes that were introduced included group poultry, piggery and horticultural interventions, which enabled livelihood diversification as well as the supplementation of staple foods [58]. Similar livelihood diversification schemes were observed by Van Riet, who noted that government-inspired hydroponics, piggery, poultry, community bakery, and food plots interventions were effective in mitigating drought effects [30]. However, the beneficiaries were too few compared to the need. Nonetheless, those who benefited had better incomes and access to food and other necessities throughout the drought seasons.

Vogel and colleagues noted that reactive state-led drought risk governance was entrenched in various policy frameworks [17]. The policies covered emergency relief and agricultural subsidies (1982/83), regional drought cooperation (1991/92), consolidated drought appeal for SADC (1994/95), joint crop assessments and early warning (1995), the establishment of country-level vulnerability assessment committees and the regional vulnerability assessment committee in 2000/01 [17]. The policies focused on food aid to reduce macro-level cereal deficits and prevent starvation at a community level. However, they were unable to proactively and holistically mitigate the effects of recurring drought. The policies were singled out as promoting dependence on humanitarian assistance and weakening resilience building because they remained delinked from development interventions [17]. The government inability to mainstream disaster management into development was corroborated by another study which noted that drought management was outsourced to consultants within the confines of assessment reports and plans for legislative compliance [30].

In a different study, Bahta and colleagues observed that inadequate government involvement negatively affected communal farmers’ drought resilience [54]. The study noted poor service delivery, insufficient and late drought relief, lack of training and lack of timely early warning information as key government failures in resilience building. It was reported that the government failed to provide security to prevent farm attacks and high stock thefts that escalated in drought periods. Inadequate government intervention aggravated drought vulnerability that was linked to psychological stress. For example, some farmers committed suicide due to the inability to cope with the drought impacts [54]. Separate studies by Akapalu and Van Riet observed stock thefts and security incidences on farms [30,61]. Drought periods corresponded with increased insecurity on farms. This was associated with aggravated unemployment and hunger, and compromised livelihoods that inhibited household capacities to adequately deal with drought effects [30,61].

An observational study conducted in northern Botswana and the Caprivi Strip of Namibia observed that government intervention with social services was critical in the adaptation to climate variability [56]. The study associated hunger, employment, human and wildlife interactions, and ill health as the main risks to livelihoods during drought. The risks were mitigated through government interventions such as wage labour, piped water, loaning farming machinery, and education and skills. The resilience and well-being outcomes observed were increased prosperity, financial stability, diversified livelihoods, access to clean water, and social change with transformed drought adaptation and coping measures [56]. Nonetheless, the study noted that some households which were still subsisting experienced two to five months of hunger and food insecurity annually.

A drought-preparedness study among Nguni cattle farmers in North-West province in South Africa observed that inadequate government support and a lack of capital, credible, and timely early warning information were the main barriers to drought preparedness [55]. There was regular contact between farmers and government extension workers, but this was never leveraged to disseminate information and educate farmers on drought mitigation. More than half of the study participants did not have drought plans, which they blamed on the inadequacy of early warning, a responsibility of the government. However, a small proportion of the farmers had fodder banks and some leased extra grazing land to mitigate the drought effects. Nonetheless, the absence of early warnings compromised farming stocks and the farmers’ socioeconomic status as they did not have alternative livelihoods. Conversely, an ethnographic study in drought-vulnerable northern Namibia credited adaptive capacity to the joint efforts of the government extension workers and the farmers [28]. The study highlighted the intrinsic success of skilled extension workers who creatively harnessed different information sources to disseminate useful and timely early warnings. The study also noted that the government was actively involved in disaster mitigation, albeit using ineffective top-down approaches that often did not recognise the different contexts and inherent capacities in the communities.

#### 3.2.2. Indigenous and Local Knowledge

Eight studies [18,28,51,52,53,57,58,62] identified indigenous and local knowledge as a determinant of resilience. They emphasised that communities with rich indigenous and local ecological knowledge and practices had good resilience outcomes than those without them for example, a study in Mogalakwena community in Limpopo, South Africa, noted that indigenous knowledge of seasons and early warnings, as well as traditional practices such as mixed cropping, the use of livestock manure, and the use of early maturing seeds, enabled the community to adapt to droughts [51]. Similarly, another study pointed out that women used indigenous knowledge and culture to promote drought adaptation behaviour [52]. Coping and adaptation practices observed were seed dressing, traditional crop maintenance, and rain-making rituals that promoted food security and a constant seed supply. Other practices noted were supplicatory rain making and communal crop protection rituals performed to invoke supernatural interventions for rain and the protection of crops from animals and birds [52].

A study of resilience in Swaziland and Lesotho observed that the application of traditional knowledge complemented with contemporary knowledge and skills improved health-related behaviour and practices [18]. It was found that knowledgeable communities were those informed about their inherent capacities and services. Such communities knew when and where to seek assistance [18]. A study of agro-ecological knowledge among Ovambo farmers in north-central Namibia suggested that mixing traditional agro-ecological knowledge with scientific agricultural knowledge co-produced hybrid knowledge without the limitations of either knowledge base [28]. The resulting hybrid agro-ecological knowledge imbued farming with drought adaptation practices such as early maturing crops, the use of donkey traction to plough large expanses of land, destocking, hunting, and gathering, and sharing food among households [28]. Interestingly, the adoption of cattle post-grazing from the predominantly traditional transhumance was noted to exacerbate land degradation.

A study of disaster-prone communities in Ngamiland District in Botswana submitted that adverse weather effects such as droughts led to a reduction in farming output and food availability, ultimately decreasing human welfare [57]. The study pointed out that the indigenous knowledge of weather forecasting (ethnometeorology) was a critical factor in adaptation and resilience. Households relied on the old tradition of observing the natural phenomena within their environment to inform their agricultural decisions. Some of the natural phenomena highlighted were the behaviour of particular plants, the presence of particular insects, wild animal migrations, and the position and brightness of particular stars. Changes in the patterns of natural phenomena symbolised good rains, poor rains, or droughts, and forewarned communities to prepare for adversity and adjust their livelihoods accordingly [57]. Similar views were observed in a study of commercial and communal farmers [62]. Farmers pointed to wind and rainfall patterns, the presence of poisonous plants in early spring, termite behaviour, and the absence of mole-mounds as drought predictors. They claimed that the observation of the natural phenomena enabled them to adjust their farming practices in anticipation of adverse weather conditions [62].

A PRA study in the Okavango Delta in north-western Botswana identified traditional community structures such as Kgotla (a traditional meeting place) and chieftaincy as strong mechanisms for mobilising adaptation to climate variability and change [53]. Traditional institutions mediated access to resources and promoted adaptation based on local ecological knowledge. Kgotla orchestrated local learning processes that empowered people to adapt or adjust their livelihoods based on the nature of adversity. Households with diversified livelihoods were cushioned from the extreme impacts of floods, human and livestock disease outbreaks, and frequent droughts. Both Kgotla and chieftaincy incentivised collective action and community participation in decision-making to mitigate periodic droughts and floods that negatively affected livelihoods and well-being. The institutions guided the development committees and volunteer associations for socio-health activities such as malaria and tsetse fly prevention. They mobilised farmer groups into traditional *malapo* (flood recession) farming which was an important livelihood in the harsh environment. Farmers adapted to planting quick maturing crops in the flood recession plains or dry river beds where soils retained moisture from seasonal floods. Traditional institutions and knowledge were credited with maintaining community cohesion and promoting adaptation to climate variability and were the central government’s heartbeat of consultation on public policy [53]. The application of traditional knowledge was also observed in a rainfall variability study in South Africa [58]. The study submitted that farmers who assimilated indigenous drought-resistant livestock breeds and crop species were able to prevent drought-induced losses [58]. However, indigenous species had lower yields but were better adapted to drought conditions than other varieties. Farmers claimed that regardless of the low yields, the crops required short growing time and reduced farming risks as well as food insecurity in drought periods. Farmers pooled their knowledge and resources to leverage scale and market opportunities which, in turn, increased their income and all-year food supply [58].

Conversely, traditional institutions and knowledge were slowly being eroded by western-modelled education and external assistance [18,28,30,53,62]. People who had acquired education undervalued traditional early warnings and some simply dismissed them in favour of scientific early warnings [53,57,62]. This was compounded by the humanitarian and development interventions by non-governmental organisations (NGOs) that inadvertently undermined intrinsic community capacities. The interventions discouraged community efforts in self-sustainment in anticipation of handouts. For example, Renzaho and colleague observed that disregard for intrinsic capacities and poor community engagement by humanitarian actors discouraged communities from anticipating and preparing for disasters [18].

#### 3.2.3. Community Capacities

Nine studies profiled the importance of community capacities in drought resilience [18,29,51,52,53,55,58,60,61]. The main elements identified were knowledge sharing, social networks, participation, cohesion, and connectedness. For example, Renzaho and colleague underscored community cohesion and support, social networks, empowerment and participation, psychological well-being and community ownership of disaster preparedness as collective elements that enhanced resilience [18]. Community cohesiveness was again identified as a resilience factor in a rainfall variability study which noted that cohesion in the community facilitated collective adaptability, including diversion from rain-fed agriculture to other livelihoods [58]. Cohesiveness and unanimity enabled the establishment of joint small-scaled commercial initiatives such as poultry and drought-resistant horticultural projects, and the establishment of a cooperative group to improve collective bargaining power and economies of scale, and address market risks [58]. Cooperation, sharing of information, costs, and risks were reported to have facilitated a drought-resilient community to emerge [58]. Similarly, a study in Thorndale in South Africa earmarked social solidarity practices such as drought committees, kinship ties, and social interaction as enhancing cohesiveness during difficult periods [61]. For example, people joined efforts to work on their community gardens where they learnt new farming ideas that they adapted in their homes. Additionally, joint efforts enabled the communities to obtain amenities such as water reservoirs and community standpipes using economies of scale.

A survey of knowledge, attitudes, and practices (KAP) in the low veld of Swaziland stated that joint community efforts led to ownership of the water infrastructure and enhanced knowledgeability and capacity to manage and maintain the infrastructure [60]. The clean water initiative attracted community participation in its functionality and maintenance. The study credited the water intervention for promoting joint learning and improvement of health outcomes such as improved hygiene and sanitation. Specifically, there was a notable increase of households with and using latrines (34.9–71.3%), and good hand-washing practices (75.5%) which were linked to hygiene and sanitation intervention by an NGO. The study associated improvements in knowledge, attitudes, and community practices in sanitation and hygiene with enhanced resilience [60]. Collective efforts were again captured in a study carried out in the Limpopo province in South Africa [51]. The study suggested that a community’s collective understanding of seasonal changes and cropping practices limited the impact of droughts on crop production and promoted adaptation. For example, short-season crops were adapted by entire communities instead of their well-liked but late maturing crops [51]. This had cascading effects on livelihoods, which also changed due to productivity and production changes in a subsistence dominated area.

A study in the Okavango Delta of Botswana found that the community management of rangelands was critical in rangeland preservation and the community’s adaptation to climate change [53]. The study highlighted that rangelands were important community resources that supported livelihoods such as livestock grazing, crafting, and hunting and gathering wild foods. Community members set, agreed on, and monitored adherence to procedures for rangelands utilisation. Rangelands were fragile and exposed to recurrent droughts and floods, as well as degradation resulting from high livestock and wildlife populations. Focusing on the nexus between people and the environment was a viable approach to risk management and the sustainability of community livelihoods and well-being in a fragile environment [53]. The relationship between rangeland management and resilience was affirmed by a 2015 study in Lesotho [29], which suggested that the poor management of the rangelands by the communities led to massive degradation and exacerbated drought effects. Specifically, the degradation led to the deterioration of crops and livestock output, which further cascaded into food insecurity and socioeconomic challenges. The study pointed to deforestation, a high old-age ratio and low education levels as key vulnerabilities in the community; these were reflected in weak coping and adaptation strategies [29].

#### 3.2.4. Household Capacities

Overwhelmingly, 16 studies submitted that household capacities were critical elements in resilience building [18,28,29,30,51,53,55,56,57,58,59,60,61,62,63,64]. The main household elements that instigated adaptation, resilience and well-being were diversified livelihoods, socioeconomic status, education levels, access to resources, and soft skills. Three studies profiled household socioeconomic status, household assets and livelihoods [29,53,56]. For example, Bunting and colleagues related household socioeconomic status to access resources [56]. The authors determined that financial assets, access to water, health and employment opportunities were the main risks to household livelihoods and well-being. Households with access to these resources coped better with adversity. The absence of or limited access to resources curtailed livelihoods, exacerbated food shortage, hunger, and poor health. For example, wage-based employability was a key factor in offsetting yearly household crop loss due to droughts and floods. Households with access to water standpipes had better water security and did not experience the burden of waterborne diseases compared to those without and those who competed with the wildlife for water sources in drought periods [56]. Likewise, Ngwenya and colleagues noted that household capital such as access to land and land use, knowledgeability, and multiple home ownership in rural and urban locations were important elements of adaptation [53]. During harsh periods, such as droughts or disease outbreaks, households escaped adversity in rural farming homes by moving to urban areas where they had homes. Moreover, the movement between areas required households to diversify livelihoods to support their well-being in either place. In rural areas, households switched land use depending on the conditions. For example, they alternated between *malapo* farming and dryland farming when the seasons changed. Correspondingly, households with more skills than farming opportunistically switched from crop production to different livelihoods such as fishing, harvesting aquatic foods, and wild plants in the delta depending on the environmental conditions. The study concluded that households’ adaptation capacities were deeply intertwined with resilience and well-being outcomes [53].

Household demographics such as gender, age, education, and socioeconomic status were strong determinants of household resilience, healthiness, and well-being [18,29,63]. For example, Belle and colleagues acknowledged that households with low education levels and socioeconomic statuses had poor coping mechanisms that perpetuated inadequacy, helplessness, and poverty [29]. The authors found that during difficult drought periods, families sent their young children into manual labour to reduce the burden of household food requirements and earn income to subsidise family needs. Another study acknowledged that cattle farming was a male’s domain and that old age was a factor in access to farming capital and grazing land [55]. The majority of the surveyed farmers were aged >60 years; youth participation was limited by the unaffordability of capital requirements and limited access to grazing land. The study further suggested that limited education among elderly farmers was a factor in the lack of drought preparedness and compromised incomes and livelihoods [55].

Similarly, Shongwe and colleagues observed in Swaziland that the ability to perceive and prepare for adversity was dependent on the age of the household [63]. The authors noted that adaptation required labour intensive agricultural activities which old people were unable to provide. Almost 62% of the household heads were aged >50 and more than 55% were illiterate, making it difficult to comprehend and apply new and improved farming systems. They continued to grow drought-intolerant crops such as maize (>90%), which further aggravated their food insecurity and health [63]. Age was also highlighted in a resilience survey that revealed elderly-headed households were less resilient to droughts than other households. Elderly-headed households also experienced weak socio-political empowerment that limited their participation and self-confidence to bring about the desired changes in their well-being [18]. Bahta and colleagues noted that gender was influential in decision making [54]. The authors observed that key decisions on drought adaptation were a male domain, with limited participation from women. Women who were responsible for food preparation did not have the bargaining power on drought-related agricultural activities. However, women were perceived to be more resilient; they knew how to search for food and ensured their children’s nutrition and well-being through drought periods [54].

Soft skills such as self-organisation, information access, ability to perceive and prepare against adversity, communication and connectedness were influential elements for household preparedness and adaptation. For example, a study of Nguni farmers attributed the lack of drought preparedness in households to inadequate access to early warning information and knowledge [55]. The regular contact between farmers and extension workers was never leveraged to disseminate information and educate farmers on drought mitigation. The lack of preparedness resulted in significant farming losses and compromised livelihoods [55]. Another study that examined the state of disaster preparedness, mitigation, and response plans found 84% and 91% of households lacked disaster preparedness plans in Lesotho and Swaziland [18]. Barriers to disaster preparedness were poor collaboration, limited resources, inability to volunteer, inability to perceive the importance of preparedness, competing priorities, poor community governance and communication, lack of funding opportunities, and dependence on humanitarian assistance. The absence of drought preparedness plans deprived households of sufficient esteem to self-determine their response and recovery strategies. It made them reliant on humanitarian assistance for survival [18].

Household connectedness and cohesion were profiled as important resilience and well-being elements. For instance, Akapalu [61] and Newsham and colleagues [28] suggested that kinship ties enabled families without access to farming land and livestock to benefit from food and livestock products from their kinsmen during difficult periods. Sharing resources through kinship ties, spousal connections and other family connectedness helped to spread drought risk across households to minimise the negative effects. Additionally, kinship ties enabled the easy flow of goods and services such as fodder, herbal medicine, and labour, which were shared among families depending on their needs [28,61].

### 3.3. Methods Used in the Development of Included Study Scales

The scales of studies included were informed by empirical studies (*n* = 5) [18,57,58,59,64] and literature review (*n* = 5) [18,29,57,59,62]. Two studies used a combination of a literature review and empirical studies (*n* = 2) [57,59]. One study (*n* = 1) was informed by a literature review, an empirical study, and the target population [57]. Interestingly, only one study (*n* = 1) combined all the four steps [18]. The remaining studies were silent on how their scales were developed (Table 2).

#### Quality Rating of Scales

We examined scales for reliability, content validity, reliability, criterion validity and construct validity, and applied a 17-point-based measure. We assessed the scales’ reliability by examining whether the test-retest and the internal consistency measured by the Cronbach Alpha were performed. Two studies (*n* = 2) [18,29] measured the internal consistency of tools. Of these, Renzaho and colleague [18] had a Cronbach Alpha ≥0.80 and was scored as good (3 points). The other study by Belle and colleague [29] had a Cronbach Alpha ≥0.764 and was scored as acceptable (2 points). The remaining studies were silent on their internal consistency.

None of the studies demonstrated having undertaken a test-retest reliability except one study (*n* = 1) [57], which suggested having undertaken it without stating details on how it was done or the outcome. We performed a criterion validity assessment to examine the relationship between the scales used and well-being outcomes. Five studies [18,54,56,59,64] showed a strong linear relationship and scored 3 points on our assessment framework informed by Cyril et al. [48] (Table 2). Two studies [60,61] showed moderate linear relationships (2 points) and three studies [57,59,62,63] were assessed to have a weak linear relationship (1 point). The rest of the studies showed no linear relationships (Table 2).

Overall, 17 psychometric properties were measured and only one study met the good criteria with a score of 10 points [18]. Three studies were assessed to be of acceptable quality (5–9 points) [29,57,60]. The remaining studies scales were assessed as poor (0–4 points) as reflected in Table 2.

### 3.4. Methodological Assessment of Studies Included

Different approaches commensurate with study types were used to assess the methodological quality of studies included. For example, we used the AACODS checklist to assess the quality of three non-peer reviewed studies included [18,61,62]. Two studies were assessed to be of high quality [18,62]. A third study was assessed to be of medium quality [61] (Table S3). The remaining 10 studies were observational and were assessed with 14-point criteria informed by the NIH checklist. One study met 11 of the 14 methodological criteria points and was assessed as good [64]. The remaining nine studies scored between 6–9 criteria points and were assessed as fair on the NIH checklist [29,54,55,56,57,58,59,60,63,64] (Table S4).

### 3.5. Quality Assessment of Qualitative Studies

Six of the studies included in the review used qualitative methods [17,28,30,51,52,53]. Their quality was assessed with a 10-point quality assessment framework informed by the Joana Briggs Qualitative Assessment and Review Instrument [37] (Table S2). Three of the studies scored between 8–10 points and were assessed as high quality [28,52,53]. The remaining three were medium quality (5–7 points) as reflected in Table S2 [17,30,51].

**Figure 3:**
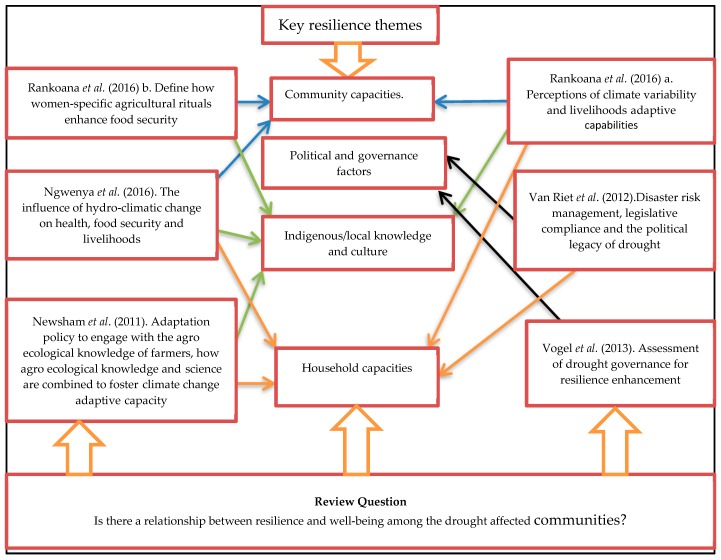
The synthesis of qualitative resilience themes adopted from Reference [46].

We conducted a thematic analysis to identify the common threads in how resilience was conceptualised in the included studies. The common resilience threads that emerged were broken into four main themes, as profiled in the summary of findings. The themes were: (i) community capacities, (ii) household capacities, (iii) indigenous/local knowledge, and (iv) political and governance capacities (Figure 3).

## 4. Discussion

Southern Africa experiences recurrent droughts that continuously erode livelihoods and affect the well-being of communities. Nonetheless, the affected communities have adopted resilience mechanisms to cope with and adapt to the periodic episodes of drought. We sought to understand the relationship between resilience to drought and well-being, examine the suitability of the resilience instruments and their psychometric properties, and identify gaps and unanswered questions in order to enhance the resilience theory in the region. We determined that 4 out of the 13 studies that applied quantitative and mixed methods [18,29,57,60] yielded acceptable to good relationships between the resilience scales and well-being (Table 2). This finding affirms that the scales used in the identified studies actually measured resilience. However, gaps in the robustness of the tools still remain, as reflected in those studies with weak or no relationships. Interestingly, all the assessed studies were silent on the construct validity of their scales, which made it difficult to determine if the scales were a result of exploratory and confirmatory analyses and whether the subscales met the minimum threshold for factor analyses. Furthermore, the internal consistency of the scales was assessed in only two studies (*n* = 2) [18,29] with good outcomes; the rest of the studies seemed not to have applied this critical measure to their scales. The lack of evidence of internal consistency in most studies suggests key gaps in the scale constructs and their subsequent outcomes. Testing the internal consistency of scales is a critical component of the accuracy and validity of data interpretation [65]. None of the qualitative studies stated their scales for us to determine their appropriateness in measuring resilience. We applied a thematic synthesis of the findings and their corresponding discussions to identify common themes as a proxy for understanding their scales. Four common themes of community capacities, household capacities, indigenous/local knowledge, and political and governance capacities emerged. Similar themes emerged from the quantitative and mixed methods studies, with an extra theme of HIV/AIDS. This finding is consistent with studies from other regions that acknowledged a combination of a few themes or individual themes with spatial variations as resilience factors [66,67]. Banding these themes together makes this study unique and provides evidence to construct a contextual composite index for benchmarking resilience interventions in southern Africa. A contextual resilience index would guide interventions to specifically apply approaches that reflect the realities of the region. In the long run, this would increase the effectiveness and efficiency of resilience interventions.

The resilience capacities at the different levels function synergistically; for example, traditional institutions of governance were critical in mobilising communities and households for adaptation to climate-induced hazards [53]. Traditional governance institutions functioned in tandem with modern governance across the region to collectively manage resources, biodiversity, and adaptation [68]. The dualism of the traditional and the modern can be further leveraged to promote hybrid knowledge, behaviour, crops, and livestock species that enhance the resilience and well-being of recurrent drought-affected people. Besides traditional knowledge pre-dating scientific knowledge, its merits in drought adaptation and resilience cannot be understated. Studies from other regions found that the rich knowledge of traditional early warnings and their application minimised disaster-induced fatalities, injuries, and livelihood losses [69,70,71]. Harnessing and blending traditional knowledge with scientific knowledge will increase understanding and acceptability among different segments of society. In addition, it will produce localised information that addresses threats to livelihoods and well-being in specific contexts. However, there is a risk of traditional knowledge disappearing or becoming redundant as more people acquire formal western education where they inadvertently get cultural miseducation. Traditional practices are indicative of people’s attachment to land and mastery of the environment. Their disappearance would lead to loss of centuries-old, rich knowledge and practices, as well as the detachment from land and the environment. Scholars have observed that detachment from land and environment aggravates its unsustainable exploitation, the loss of a community’s adaptation capacities, and poor well-being outcomes [72,73,74].

Humanitarian interventions to mitigate disasters focused on welfare, a costly approach that provided only temporary relief [18,75]. While such interventions assist households to cope, they do not necessarily enhance resilience [14]. Our findings suggest multi-sectoral poverty alleviation interventions reinforced household and community level capacities to create resilient communities. This challenges the findings from other regions that confined resilience and well-being to household and community level capacities [76,77,78,79]. We advance that household level capacities work in tandem with community and governance level capacities to grow resilience and advance well-being outcomes.

Interestingly, only one study included in the review associated HIV/AIDS with droughts, yet evidence suggests that southern Africa has one of the world’s highest HIV/AIDS burdens [80]. This mismatch suggests that drought-resilience interventions may be missing the nexus between the burden of HIV/AIDS and drought resilience. Addressing the burden of HIV/AIDS in the context of drought resilience would go a long way in reducing vulnerabilities and enhancing the well-being of households. It would also create a synergistic and comprehensive resilience framework that optimises resources and well-being outcomes.

### Limitations

Firstly, the variability of study designs made meta-analysis inapplicable to this review. Secondly, we used common quality assessment tools that focus on specific areas of quality that could have differed from the aims and objectives of included studies. Thirdly, the generalisability and transferability of the findings beyond the study area should be taken with caution because our study parameters were restricted to southern Africa. We could have inadvertently excluded eligible studies that were not accessible, so only studies available and accessible were included in this review.

## 5. Policy Implications

This synthesis of evidence to identify the relationship between resilience to drought and health and well-being is timely and will assist to stimulate appropriate planning and policy interventions. Moreover, because the evidence suggests that there is strong likelihood of continuous occurrence of drought-induced disasters across the region. This calls for a serious review and update of regional and national disaster management policies which mostly aim to resist and/or mitigate disaster effects. New and revised policies that focus on the transformation of communities’ capabilities and equip them in such a way as to cope and adapt to recurring drought conditions are urgently needed. Such policies should perceive resilience as a culture and a way of life just like droughts are quickly becoming. To achieve this, further research is necessary to establish the regional context specific resilience measurement constructs that will benchmark resilience policies and interventions. Such measurement constructs can be yielded from nurturing and supporting local-led research initiatives to ensure the outcomes are evidence-driven and not just transplanted from other regions without validation. There is an urgent need for a regional resilience institute to continuously generate evidence to inform regional and national policies. Such a structure will significantly reduce the cost and burden of droughts on the regional economies, agriculture, and people.

Additionally, there is need for policymakers to have clarity on the difference between resilience and disaster risk reduction interventions. Disaster risk reduction deals with the identification of hazards, analysis of hazard impacts and causes, and the removal or reduction of vulnerabilities [81]. On the contrary, resilience deals with the transformation of people’s capacities to cope, overcome, and recover from disaster effects. Differentiating between the two would generate a paradigm shift and re-orient disaster governance from the conventional disaster management in the region to a new culture of resilience building. Equally important is the need for the external assistance to be focussed on boosting inherent resilience capacities rather than the periodical emergency responses.

## 6. Conclusions

Droughts remain a serious threat to well-being, and cannot be effectively stopped. However, peoples’ capacities to cope, adapt, and live with it can be improved. This systematic review highlights the critical factors that can improve and unlock people’s capacities to live with droughts. Our evidence suggests that resilience capacities are vested in households, communities, and at a government level. Poverty alleviation policies were important in strengthening resilience and well-being outcomes. Equally important was the need to leverage blended traditional knowledge with modern scientific knowledge to enhance resilience and optimise well-being outcomes. However blending traditional and scientific knowledge and processes remains a research gap, which, when addressed, will create a wider pool of disaster resilience information and knowledge. Additionally, it will preserve centuries of old traditions and increase acceptability and uptake by the segments of society.

We suggest that age, gender, race, and diversified livelihoods were central in mediating access to resources that unlocked household and community resilience. However, persistent drought-induced stress, food insecurity, hunger, age, racialised farming, and insecurity on farms undermined people’s well-being. The need for resilience policies and interventions to focus on these key aspects cannot be understated.

## Figures and Tables

**Table 1:** The study characteristics.

Author (Year) [Ref]	Study Design & Data Collection	Sample Characteristics	Study Setting	Study Objectives	Main Resilience Determinants	Assessment Outcome
Rankoana (2016) [51]	A qualitative study using open-ended questions.	*n* = 100 participants Male = 52; Female = 48	Drought prone Mogalakwena community in the Limpopo province of South Africa	Explore community perceptions of climate variability and the capabilities to adapt livelihoods	Household capacities, community capacities, and Indigenous/local knowledge	Medium (JBI QARI)
Van Riet (2012) [30]	A qualitative control case study of black communal and white commercial farmers using the Mmogo method.	*n* = 37 participants (FGD = 7), KIs (farmers = 4, State officials = 7), Farmers aged <60 years	Drought-prone North-West province, South Africa	Disaster risk management, legislative compliance, and accompanying political legacy	Political and governance capacities, and household capacities	High (JBI QARI)
Vogel et al. (2010) [17]	A retrospective longitudinal study using secondary data (policy reports) supplemented by primary data collection.	*n* = 27 subject matter expert interviews and focus group discussions (*n* = not given).	Subject matter experts working within the SADC regional bodies, commercial farmers, and members of the African Farmers Union	Assessment of drought governance for resilience enhancement	Political and governance capacities	High (JBI QARI)
Rankoana (2016) b [52]	A qualitative study through focus group discussions.	*n* = 50 participants Age = 35–78 years	Dikgale community in Limpopo, South Africa	Define how agricultural women-specific rituals enhance food security	Community capacities and Indigenous/local knowledge	High (JBI QARI)
Ngwenya et al. (2016) [53]	A participatory rural appraisal (PRA). Additional data was sourced through field observations, unstructured interviews and secondary data.	*n* = 18 FGDs. The FGD groups comprised of males only = 3 groups, females only = 3 groups, combined males and females = 12 groups	Farming communities in Okavango Delta, Botswana	Examine the influence of hydro-climatic change on health, food security, and livelihoods	Community capacities; Indigenous/local knowledge; household capacities	High (JBI QARI)
Newsham et al. (2011) [28]	An ethnographic study of knowledge, farming, and climate change adaptation.	*n* = 8 FGDs. Each FGD comprised of 10–15 participants. Farmers and extension workers	Rural drought-prone Omusati region of north-west Namibia	Establish the need for adaptation policy to engage with the agro-ecological knowledge of farmers; capture how agro-ecological knowledge and science are combined to foster a climate change adaptive capacity	Household capacities; Indigenous/local knowledge; and political and governance capacities	Medium (JBI QARI)
Renzaho et al. (2016) [18]	Mixed methods with non-equivalent control groups’ post-test only quasi-experimental design. Qualitative data collection through FGD. Quantitative data collection using a structured questionnaire and systematic sampling.	Qualitative data involved 16 FGDs, *n* = 197 (93 in Swaziland and 104 in Lesotho). For quantitative data, *n* = 3324 households (1789 in Swaziland and 1535 in Lesotho)	Drought-prone farming households in rural Lesotho and Swaziland	Examine resilience to droughts and develop an evidence-based framework to inform community resilience interventions	Household capacities, community capacities; and Indigenous/local knowledge	High (AACODS)
Bahta et al. (2016) [54]	Cross-sectional survey. Data collection using a semi-structured questionnaire, purposive sampling method procedures, and the creation of a perception index.	*n* = 87 participants Male = 62 Female = 25 Mean age = 51 years	Communal farmers in OR Tambo district, Eastern Cape province in South Africa	Examine farmers’ awareness of drought, their vulnerabilities and relationships with gender, networks, stress, security and the role of government	Governance capacities and community capacities	Fair (NIH)
Bareki et al. (2017) [55]	Mixed methods. Qualitative data collection through in-depth face to face interviews. Quantitative data collection using a semi-structured questionnaire.	*n* = 85 participants	Nguni cattle development project members in North-West province in South Africa	Assess drought preparedness of intervention beneficiaries and identify factors of drought-preparedness among Nguni cattle farmers	Governance capacities, and household capacities	Fair (NIH)
Bunting et al. (2013) [56]	Cross-sectional survey Data collection using semi-structured open-ended questionnaires, convenience sampling.	*n* = 330 households	Households in seven arid/semi-arid villages across the Okavango, Kwando and Zambezi catchments in Botswanan and Namibia	Explore perceptions of livelihood risk in the semi-arid Savanah and Zambezi catchments and how perceived risk mirrors the changing ecosystem in Botswana and Namibia	Governance capacities and household capacities	Fair (NIH)
Kolawole et al. (2016) [57]	Mixed methods. Qualitative data collection through key informant interviews, FGDs, and a stakeholder workshop. Quantitative data collection using a closed-end questionnaire. A multi-stage sampling procedure was used.	*n* = 592 households 27 FGDs Mean age = 51 years	Eight rural communities in the Okavango Delta in the Ngamiland district of Botswana	Investigate the impacts of climate variability on agriculture and identify adaptation strategies	Indigenous/local knowledge and household capacities	Fair (NIH)
Belle et al. (2015) [29]	A mixed methods cross-sectional survey.	Household survey (*n* = 102); KIs (*n* = 3)	Subsistence farmers in the drought-prone Koiti-Se-Phola community, Mafeteng district of Lesotho	Investigate the community’s vulnerability to agricultural drought to inform resilience building	Household capacities and community capacities	Fair (NIH)
Thomas et al. (2007) [58]	A mixed methods observational study base on secondary rainfall data using Self-Organising Maps (SOMs) and primary data gathered through FGDs and KIs.	Secondary rainfall data Primary data: FGDs (*n* = 50); KIs (*n* = 30)	Natural resource dependent communities in three regions of Limpopo, KwaZulu Natal and north-west provinces	Analyse rainfall variability, the community’s awareness of the variability and their adaptive capacities	Household capacities and community capacities, and indigenous/local knowledge	Fair (NIH)
Mlenga et al. (2015) [59]	Mixed methods. Qualitative data collection through structured and unstructured interviews and FGDs. Quantitative data collection using a questionnaire. A random sampling technique was used.	*n* = 200 households	Drought-vulnerable farming households that benefited from NGO climate change and drought mitigation interventions in the Lowveld agro-ecological zone of Swaziland	To understand the determinants of conservation agriculture (CA) in the Lowveld agro-ecological zone of Swaziland	Household capacities	Fair (NIH)
Mlenga et al. (2016) [60]	A knowledge, attitudes, and practices (KAP) survey conducted in the low veld agro-ecological zone of Swaziland.	*n* = 450	Drought-prone beneficiaries of a water sanitation and hygiene (WASH) intervention	Evaluate the effectiveness of a WASH intervention in mitigating disaster risk and enhancing community resilience	Household capacities and community capacities and health	Fair (NIH)
Akpalu (2005) [61]	Mixed methods. Qualitative data collection through an open-ended questionnaire. Quantitative data collection using closed-ended questionnaire. A random sampling technique was used.	*n* = 34 participants Male = 10 Female = 24 Age = 26–85 years	Drought-affected Thorndale located in the Bushbuckridge region of the Limpopo province in South Africa	Assess the effects of the 2002/2003 drought, the responses, constraints encountered and the implications of the drought on HHs	Household capacities and community capacities	Medium (AACODS)
Hudson (2002) [62]	Mixed methods. Qualitative data collection through interviews. Quantitative data collection using questionnaires.	Commercial farmers (*n* = 25); Communal farmers (*n* = 35)	Commercial and communal livestock farmers in the North-West province in South Africa	Assess and compare commercial and communal livestock farmers’ drought management strategies	Political and governance capacities, and household capacities	High (AACODS)
Shongwe et al. (2014) [63]	A cross-sectional study Quantitative data collection through questionnaires.	*n* = 350	Rain-dependent farming households on Swazi communal land in Mpolojeni in the Lowveld of Swaziland.	Identify household adaptation strategies and determinants of the choice of strategies	Household capacities and community capacities	Fair (NIH)
Mason (2005) [64]	An analysis of secondary epidemiological data drawn from national and subnational surveys such as demographic health surveys (DHS) and multiple indicator cluster surveys (MICS) in southern Africa.	Secondary anthropometric and HIV prevalence data drawn from six countries with UNICEF support	Children of 0–5 years in Lesotho and Swaziland	Explore child malnutrition trends in relation to HIV/AIDS and the 2001–2003 drought	Household capacities and health factors	Good (NIH)

**Table 2:** The quality assessment of scales.

Author Year [Ref]	Content Validity				Reliability		Criterion Validity	Construct Validity (EFA and/or CFA)				Total Points
	Informed by Literature Review	Panel of Experts	Empirical Study	Reviewed by Target Population	Internal Consistency	Test-Retest		Factors Explained ≥50% of the Variance	Included at Least 3 Items	Variables Loading	Based on 10 Cases per Variable	
	Yes = 1 No = 0	Yes = 1 No = 0	Yes = 1 No = 0	Yes = 1 No = 0	0–3 Points	0–3 Points	0–3 Points	Yes = 1 No = 0	Yes = 1 No = 0	Yes = 1 No = 0	Yes = 1 No = 0	Maximum Points = 17
Renzaho et al., 2016 [18]	1	1	1	1	3	0	3	0	0	0	0	10
Bahta et al., 2016 [54]	0	0	0	0	0	0	3	0	0	0	0	3
Bareki et al., 2017 [55]	0	0	0	0	0	0	0	0	0	0	0	0
Bunting et al., 2013 [56]	0	0	0	0	0	0	3	0	0	0	0	3
Kolawole et al., 2016 [57]	1	0	1	1	0	1 *	1	0	0	0	0	5
Belle et al., 2015 [29]	1	1	0	0	2	0	2	0	0	0	0	6
Thomas et al., 2007 [58]	0	0	1	0	0	0	2	0	0	0	0	3
Mlenga et al., 2015 [59]	0	0	0	0	0	0	1	0	0	0	0	1
Mlenga et al., 2016 [60]	1	0	1	0	0	0	3	0	0	0	0	5
Akpalu et al., 2005 [61]	0	0	0	0	0	0	0	0	0	0	0	0
Hudson et al., 2002 [62]	1	0	0	0	0	0	1	0	0	0	0	2
Shongew et al., 2014 [63]	0	0	0	0	0	0	1	0	0	0	0	1
Mason 2005 [64]	0	0	1	0	0	0	3	0	0	0	0	4

* Denotes item mentioned in the report without stating details.

## Supplementary Materials

The following are available online at http://www.mdpi.com/1660-4601/15/11/2375/s1, Table S1: The PRISMA checklist, Table S2: Quality assessment of qualitative studies based on JBIQARI, Table S3: Quality assessment for grey literature based on AACODS, Table S4: Quality of peer-reviewed studies included based on NIH quality assessment checklist, Table S5: Rating of scales based on framework by Cyril and colleagues.
