# Supplementary material for: Resilience to Climate-Induced Disasters and Its Overall Relationship to Well-Being in Southern Africa: A Mixed-Methods Systematic Review
Source: Int J Environ Res Public Health. 2018 Oct 26;15(11):2375. doi: 10.3390/ijerph15112375 (PMC6267582; doi:10.3390/ijerph15112375)
Supplement: Supplementary file 1 [file ijerph-15-02375-s001.pdf]

Table S1. The PRISMA checklist.

PRISMA 2009 Checklist

Section/topic	#	Checklist item	Reported on page #
**TITLE**			
Title	1	Identify the report as a systematic review, meta-analysis, or both.	1
**ABSTRACT**			
Structured summary	2	Provide a structured summary including, as applicable: background; objectives; data sources; study eligibility criteria, participants, and interventions; study appraisal and synthesis methods; results; limitations; conclusions and implications of key findings; systematic review registration number.	1
**INTRODUCTION**			
Rationale	3	Describe the rationale for the review in the context of what is already known.	2-3
Objectives	4	Provide an explicit statement of questions being addressed with reference to participants, interventions, comparisons, outcomes, and study design (PICOS).	4
**METHODS**			
Protocol and registration	5	Indicate if a review protocol exists, if and where it can be accessed (e.g., Web address), and, if available, provide registration information including registration number.	5
Eligibility criteria	6	Specify study characteristics (e.g., PICOS, length of follow-up) and report characteristics (e.g., years considered, language, publication status) used as criteria for eligibility, giving rationale.	7
Information sources	7	Describe all information sources (e.g., databases with dates of coverage, contact with study authors to identify additional studies) in the search and date last searched.	6
Search	8	Present full electronic search strategy for at least one database, including any limits used, such that it could be repeated.	6
Study selection	9	State the process for selecting studies (i.e., screening, eligibility, included in systematic review, and, if applicable, included in the meta-analysis).	7
Data collection process	10	Describe method of data extraction from reports (e.g., piloted forms, independently, in duplicate) and any processes for obtaining and confirming data from investigators.	7- 8
Data items	11	List and define all variables for which data were sought (e.g., PICOS, funding sources) and any assumptions and simplifications made.	7
Risk of bias in individual studies	12	Describe methods used for assessing risk of bias of individual studies (including specification of whether this was done at the study or outcome level), and how this information is to be used in any data synthesis.	8-9
Summary measures	13	State the principal summary measures (e.g., risk ratio, difference in means).	N/A
Synthesis of results	14	Describe the methods of handling data and combining results of studies, if done, including measures of consistency (e.g., I^2) for each meta-analysis.	9-10

PRISMA 2009 Checklist

Section/topic	#	Checklist item	Reported on page #
Risk of bias across studies	15	Specify any assessment of risk of bias that may affect the cumulative evidence (e.g., publication bias, selective reporting within studies).	8-9
Additional analyses	16	Describe methods of additional analyses (e.g., sensitivity or subgroup analyses, meta-regression), if done, indicating which were pre-specified.	N/A
**RESULTS**			
Study selection	17	Give numbers of studies screened, assessed for eligibility, and included in the review, with reasons for exclusions at each stage, ideally with a flow diagram.	5
Study characteristics	18	For each study, present characteristics for which data were extracted (e.g., study size, PICOS, follow-up period) and provide the citations.	11-14
Risk of bias within studies	19	Present data on risk of bias of each study and, if available, any outcome level assessment (see item 12).	8-9
Results of individual studies	20	For all outcomes considered (benefits or harms), present, for each study: (a) simple summary data for each intervention group (b) effect estimates and confidence intervals, ideally with a forest plot.	16-25
Synthesis of results	21	Present results of each meta-analysis done, including confidence intervals and measures of consistency.	N/A
Risk of bias across studies	22	Present results of any assessment of risk of bias across studies (see Item 15).	9-10
Additional analysis	23	Give results of additional analyses, if done (e.g., sensitivity or subgroup analyses, meta-regression [see Item 16]).	N/A
**DISCUSSION**			
Summary of evidence	24	Summarize the main findings including the strength of evidence for each main outcome; consider their relevance to key groups (e.g., healthcare providers, users, and policy makers).	16-25
Limitations	25	Discuss limitations at study and outcome level (e.g., risk of bias), and at review-level (e.g., incomplete retrieval of identified research, reporting bias).	30-31
Conclusions	26	Provide a general interpretation of the results in the context of other evidence, and implications for future research.	32
**FUNDING**			
Funding	27	Describe sources of funding for the systematic review and other support (e.g., supply of data); role of funders for the systematic review.	34

From: Moher D, Liberati A, Tetzlaff J, Altman DG, The PRISMA Group (2009). Preferred Reporting Items for Systematic Reviews and Meta-Analyses: The PRISMA Statement. PLoS Med 6(7): e1000097. doi:10.1371/journal.pmed1000097

For more information, visit: www.prisma-statement.org.

Table S2. Quality assessment of qualitative studies based on JBIQARI.

Criteria	Author [Year]					
	**Rankoana et al. (2016)a [51]**	**Van Riet et al. (2012) [52]**	**Vogel et al. (2010) [13]**	**Rankoana et al. (2016)b [53]**	**Ngwenya et al. (2016) [54]**	**Newsham et al. (2011) [55]**
Is there congruity between the stated philosophical perspective and the research methodology?	Yes	Yes	Yes	Yes	Yes	Yes
Is there congruity between the research methodology and the research question or objectives?	Yes	Yes	Yes	Yes	Yes	Yes
Is there congruity between the research methodology and the methods used to collect data?	Yes	Yes	Yes	Yes	Yes	Yes
Is there congruity between the research methodology and the representation and analysis of data?	Yes	Yes	Yes	Yes	Yes	C/D
Is there congruity between the research methodology and the interpretation of results?	Yes	Yes	Yes	Yes	Yes	Yes
Is there a statement locating the researcher culturally or theoretically?	No	No	Yes	Yes	No	Yes
Is the influence of the researcher on the research, and vice- versa, addressed?	C/D	Yes	No	Yes	Yes	No
Are participants, and their voices, adequately represented?	No	Yes	Yes	Yes	Yes	Yes
Is the research ethical according to current criteria or, for recent studies, and is there evidence of ethical approval by an appropriate body?	Yes	No	No	No	No	No
Do the conclusions drawn in the research report flow from the analysis, or interpretation, of the data?	Yes	Yes	Yes	Yes	Yes	Yes
Overall points<br>Quality rating	**7**<br>**Medium**	**8**<br>**High**	**8**<br>**Medium**	**9**<br>**High**	**8**<br>**High**	**7**<br>**Medium**

N/A = Not Applicable; CD = Cannot Determine; Yes = 1 point; No = 0 point; N/A = 0 point; CD = 0 point; Rating: High = 8-10 points; Medium = 5-7 points; Low = <4 points

Table S3. Quality assessment for grey literature based on AACODS.

Dimension	Criteria	Author [Year]		
		**Renzaho et al. (2016) [14]**	**Akpalu et al. (2005)[64]**	**Hudson et al. (2002)[65]**
Authority	**Individual author**			
	Author associated with a reputable organisation	Yes	No	No
	Author has professional qualification or considerable experience	Yes	No	No
	Author has produced or published other work in the field	Yes	No	No
	Author is a recognised expert, identified in other sources	Yes	No	No
	Cited by others	Yes	No	No
	Is a higher degree student under expert supervision	N/A	Yes	Yes
	**Host Institution**			
	Host institution is repeatable	Yes	Yes	Yes
	Host institution is an authority in the field	Yes	Yes	Yes
	**All cases**			
	Detailed referencing list or bibliography	Yes	Yes	Yes
	**Dimension points scored**	**8/9**	**3/9**	**3/9**
Accuracy	Item has clearly stated aim or brief	Yes	Yes	Yes
	If so, was the aim met?	Yes	Yes	Yes
	Has stated methodology	Yes	Yes	Yes
	Methodology was adhered to	Yes	Yes	Yes
	Has been peer-reviewed	No	No	No
	Edited by a reputable authority	Yes	No	C/D
	Supported by authoritative, documented references or credible sources	Yes	Yes	Yes
	Is representative of work in the field; if not is it a valid counterbalance?	Yes	No	No
	Data collection is explicit and appropriate for the research	Yes	No	Yes
	If item is secondary material (e.g. policy brief or technical report), does it refer to original?	N/A	N/A	N/A
	Accurate and unbiased interpretation or analysis	Yes	No	Yes
	**Dimension points scored**	**9/11**	**5/11**	**7/11**
Coverage	Refers to a particular population, designed to answer a particular question or based on statistics from a particular survey with limits clearly stated	Yes	Yes	Yes

	**Dimension points scored**	**1/1**	**1/1**	**1/1**
Objectivity	Has clarity of author's stand point	Yes	No	Yes
	Work balanced in presentation	Yes	No	Yes
	**Dimension points scored**	**2/2**	**0/2**	**2/2**
Date	Study date clearly stated or can be ascertained	Yes	No	Yes
	Includes contemporary material	Yes	Yes	Yes
	**Dimension points scored**	**2/2**	**1/2**	**2/2**
Significance	Item is meaningful (incorporates feasibility, utility and relevance)	Yes	Yes	Yes
	Does it add to context?	Yes	Yes	Yes
	Enriches or adds something unique to research	Yes	Yes	Yes
	Strengthens or refutes current position	Yes	Yes	Yes
	Research area would be lesser without it	Yes	C/D	Yes
	Is it integral, representative or typical?	Yes	Yes	Yes
	Has impact (influential to others' work or behaviour)	Yes	C/D	Yes
	**Dimension points scored**	**7/7**	**5/7**	**7/7**
Total points<br>Rating		**29/32**<br>**High**	**15/32**<br>**Medium**	**22/32**<br>**Medium**

N/A = Not Applicable; CD = Cannot Determine; Yes = 1 point; No = 0 point; N/A = 0 point; CD = 0 point; Rating: High = >24-32 points; Medium= 15-22 points; Low= <14 points

Table S4. Quality of peer-reviewed studies included based on NIH quality assessment checklist.

Criteria	Author [Year]									
	**Bahta et al. (2016) [56]**	**Bareki et al. (2017) [57]**	**Bunting et al. (2013) [58]**	**Kolawole et al. (2016) [59]**	**Belle et al. (2015) [60]**	**Thomas et al. (2007) [61]**	**Mlenga et al. (2015) [62]**	**Mlenga et al. (2016) [63]**	**Shongwe et al. (2014) [66]**	**Mason et al. (2005) [67]**
Study objectives or research question clearly stated	Yes	Yes	CD	Yes	Yes	Yes	Yes	Yes	Yes	Yes
Study population clearly specified and defined	Yes	Yes	Yes	Yes	Yes	Yes	Yes	Yes	Yes	Yes
Participation rate of eligible persons at least 50%?	Yes	Yes	Yes	Yes	Yes	No	Yes	Yes	Yes	Yes
Participants selected or recruited from the same or similar populations (including the same time period?) Were inclusion and exclusion criteria for being in the study pre-specified and applied uniformly to all participants?	Yes	Yes	Yes	Yes	Yes	No	Yes	Yes	Yes	Yes
Was a sample size justification, power description, or variance and effect estimates provided?	No	No	CD	Yes	Yes	No	Yes	Yes	Yes	CD
Exposure(s) measured before the outcomes(s) being measured	No	No	No	No	No	Yes	No	No	No	Yes
Time frame sufficient to observe association between exposure and outcome	No	No	No	No	No	Yes	No	No	No	Yes
For the exposure, did the study examine different levels of the exposure as related to the outcome	No	No	Yes	Yes	Yes	Yes	Yes	Yes	Yes	Yes
Exposure measures (Independent variables) clearly defined, valid, reliable, and implemented consistently across all study participants	Yes	Yes	Yes	Yes	Yes	Yes	Yes	Yes	Yes	Yes
Exposure(s) measured more than once over the time	N/A	N/A	N/A	N/A	N/A	Yes	N/A	N/A	N/A	Yes
Outcome measures (dependent variables) clearly defined, valid, reliable, and implemented consistently across all study participants	Yes	Yes	Yes	Yes	Yes	Yes	Yes	Yes	Yes	Yes
Outcome assessors blinded to the exposure status of participants	N/A	N/A	N/A	N/A	N/A	N/A	N/A	N/A	N/A	N/A
Loss to follow-up after baseline 20% or less	N/A	N/A	N/A	N/A	N/A	N/A	N/A	N/A	N/A	CD
Potential confounding variables measured and adjusted statistically for their impact on the relationship between exposure(s) and outcome(s)	N/A	No	Yes	Yes	Yes	No	Yes	No	Yes	Yes
Overall points	6	6	7	9	9	8	9	8	9	11
Quality rating	Fair	Fair	Fair	Fair	Fair	Fair	Fair	Fair	Fair	Good

N/A = Not Applicable; CD = Cannot Determine; Yes = 1 point; No = 0 point; N/A = 0 point; CD = 0 point; Rating: Very good = >13 points; Good = 10-13 points; Fair = 5 – 9 points; Poor = <4 points.

Table S5. Rating of scales based on framework by Cyril and colleagues.

Content Validity				Reliability		Criterion validity	Construct validity	Total Psychometric properties
Was the tool development informed by:			**Tool reviewed by target population**	**Internal consistency** (Cronbach's alpha)	**Test-retest** (intra-class correlation coefficient)		**EFA & CFA**	**Total maximum score =17**
Literature review	**Panel of experts**	**Empirical study**						
yes=1 point	yes=**1 point**	yes=**1 point**	yes=**1 point**	<0.50=unacceptable **(0 point)**	<0.40—poor. **(0 point)**	no linear relationship **(0 point)**	Extracted factors explained ≥50% of the variance **(yes=1 point, no=0 point)**	0-4 points= poor
no=0 point	no=**0 point**	no=**0 point**	no=**0 point**	≥0.50 and <0.70 =poor **(1 point)**	≥0.40 and <0.60=fair **(1 point)**	0.30=a weak linear relationship **(1 point)**	each extracted factor has at least 3 items **(yes=1 point, no=0 point)**	5-9 points acceptable
				≥70 and <0.80= acceptable **(2 points)**	≥0.60 and <0.75= good **(2 points)**	0.50=a moderate relationship **(2 points)**	each variable loads strongly on only one factor (≥0.35) and has two or more strong loadings (≥0.70) **(yes=1 point and no=0 point)**	10-13 points good
				≥0.80=good **(3 points).**	≥75= very good **(3 points)**	≥0.70=a strong linear relationship **(3 points)**	factor analysis was based on at least 10 cases per variable **(yes=1 point and no= 0 point)**	>13 very good
